# Appendiceal mucinous neoplasm in adolescence: Diagnosis, management, and surveillance

**DOI:** 10.1002/jpr3.12017

**Published:** 2023-12-27

**Authors:** Andrew Farrar, Paola Blanco, Abigail Fabbrini, Lindsey Armstrong, Jonathan Metts, Hector Monforte, Sara Karjoo, Paul Danielson, Michael Wilsey

**Affiliations:** ^1^ College of Osteopathic Medicine Kansas City University Kansas City Missouri USA; ^2^ Department of Pediatrics Morsani College of Medicine, University of South Florida Tampa Florida USA; ^3^ Division of Surgery Advent Health for Children Orlando Florida USA; ^4^ Hematology/Oncology Johns Hopkins All Children's Hospital Saint Petersburg Florida USA; ^5^ Anatomic Pathology Johns Hopkins All Children's Hospital Saint Petersburg Florida USA; ^6^ Pediatric Gastroenterology Johns Hopkins All Children's Hospital Saint Petersburg Florida USA; ^7^ Division of Surgery Johns Hopkins All Children's Hospital Saint Petersburg Florida USA

**Keywords:** appendix, myxoglobulosis, pediatric, pseudomyxoma peritonei, tumor

## Abstract

This case report describes a 17‐year‐old patient with a low‐grade appendiceal mucinous neoplasm. The patient presented with non‐bloody diarrhea, abdominal pain, and weight loss. A colonoscopy revealed a cecal polypoid mass that required laparoscopic surgery. The residual appendix was dilated with myxoglobulosis and histopathology confirmed the diagnosis of a low‐grade appendiceal mucinous neoplasm staged pT3Nx. The potential risk of pseudomyxoma peritonei is a serious complication of these tumors. Surveillance plans include computed tomography abdomen and pelvis, and tumor markers every 6 months for the next 2 years. This case highlights the importance of considering appendiceal malignancy in patients with abdominal pain and weight loss, despite the rarity of the disease. It also emphasizes the need for careful monitoring due to the possible complications associated with these tumors. Treatment and prognosis for appendiceal neoplasms depend on the histopathologic characteristics, tumor‐nodes‐metastasis stage, tumor grade, and presence of peritoneal disease.

## 1 INTRODUCTION

Appendiceal mucinous neoplasms are exceedingly rare tumors. They account for 0.4%–1% of all gastrointestinal malignancies in the United States and have an age‐adjusted, yearly incidence of about 0.12 cases per 1 million individuals.^1^ These lesions are extremely uncommon in the pediatric population. Most are diagnosed in adults in their fifth or sixth decade of life, and there is a slight female predilection.^1^, ^2^, ^3^ Low‐grade appendiceal mucinous neoplasms are typically an incidental finding during work‐up for suspected appendicitis, adnexal masses, or other retroperitoneal tumors.^2^, ^4^

Patients are often asymptomatic or present with nonspecific symptoms. Symptoms may include right lower quadrant pain, weight loss, rectal bleeding, or changes in bowel habits. In several studies, the most common symptom was abdominal pain.^2^, ^4^ Serious complications associated with appendiceal mucinous neoplasms include intestinal obstruction, intestinal perforation, mucocele rupture, and pseudomyxoma peritonei (PMP).^3^

### 1.1 Case report

**Figure 1:**
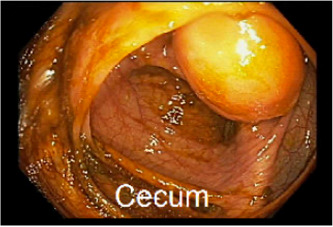
A large 2.5 × 2.5 × 2.4 cm cecal polypoid mass in the cecum.

**Figure 2:**
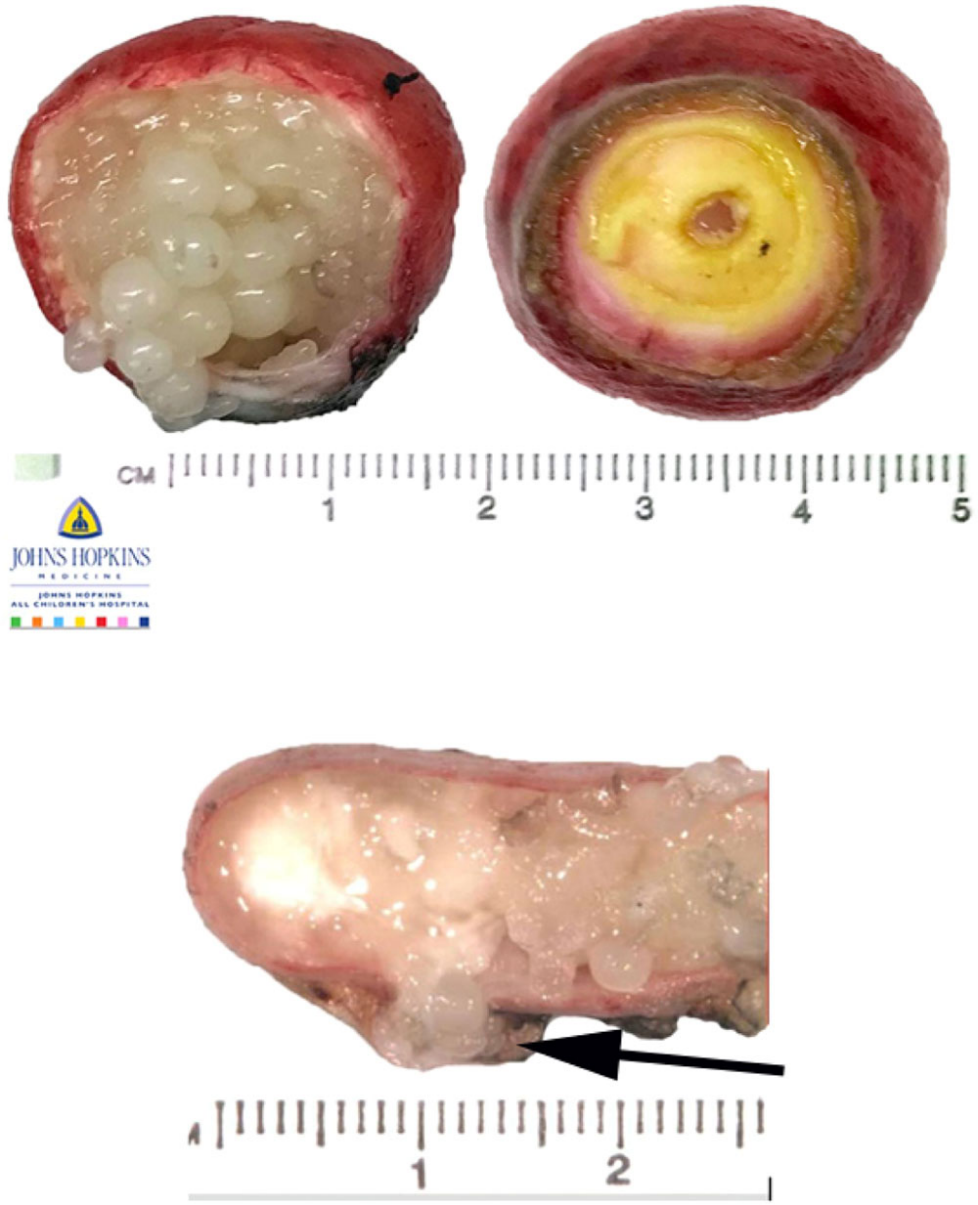
Cecal mass cut (left) base with proximal opening of appendix orifice at base (right) and appendix specimen tip (bottom) showing mucin globule concretion filling mucosa‐covered cecal protruding mass and appendiceal lumen, note mucin seepage outside wall in appendix tip (arrow).

**Figure 3:**
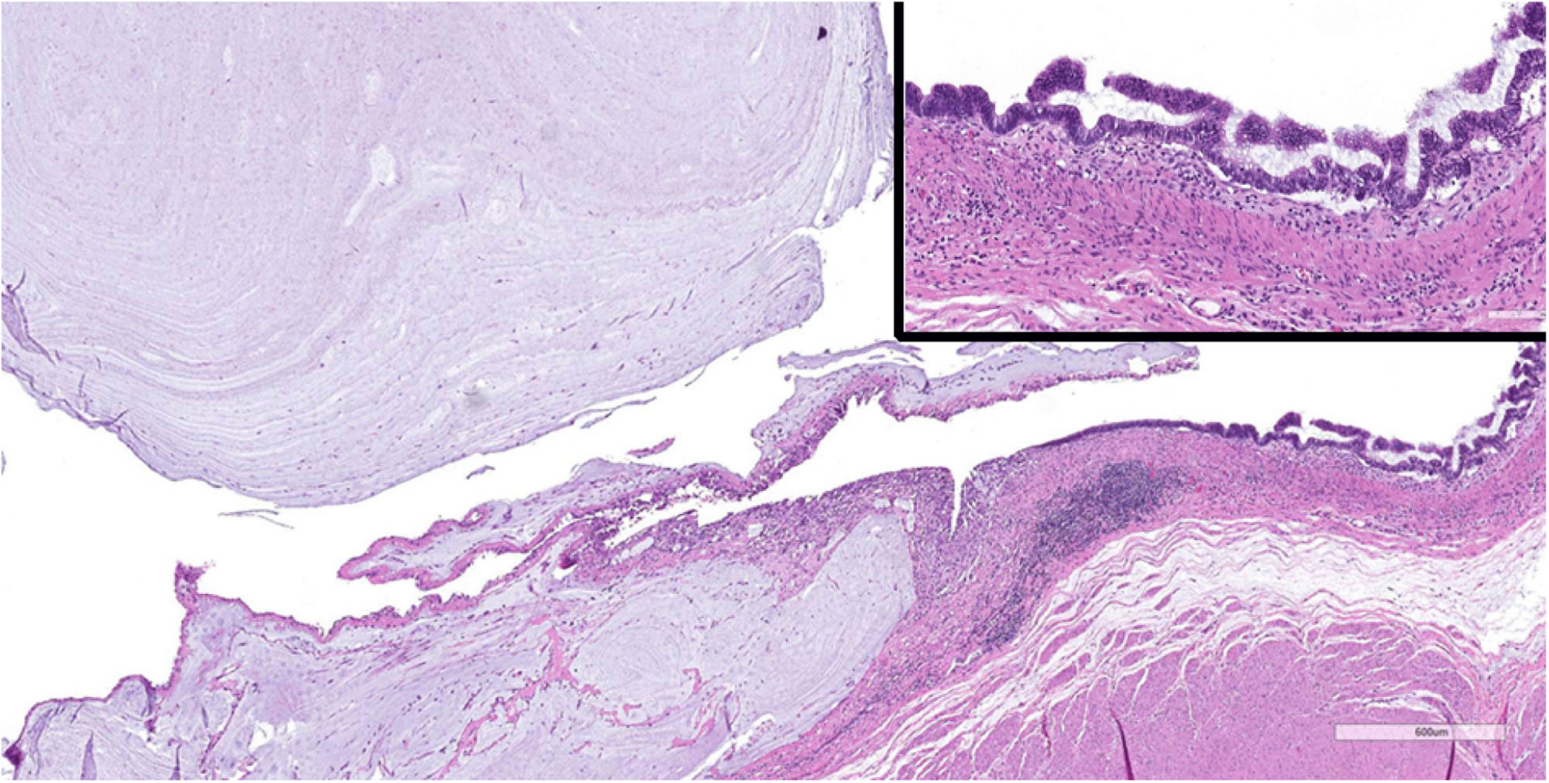
Histopathology H&E section of distal appendix tip focally lined by dysplastic atypical mucin producing epithelium and lumen filled with acellular mucin seeping into submucosa and rounded globules in lumen. Insert (upper right) higher magnification of lining epithelium, no cellular invasion of wall was noted.

A 17‐year‐old male with a family history of Crohn's disease presented to the pediatric gastroenterology clinic with a 9‐month history of nausea, lower abdominal pain, non‐bloody diarrhea, and a 20‐pound weight loss. The pain was described as constant pressure and stabbing. There were no associated nighttime awakenings. The diarrhea was watery, occurring up to five times per day. Laboratory testing, screening stool studies, and upper endoscopy were unremarkable. However, a large 2.5 × 2.5 × 2.4 cm cecal polypoid mass was visualized during the colonoscopy (Figure 1) and resected endoscopically, resulting in a subsequent small cecal perforation that could not be closed due to the location. During laparoscopic examination, the residual appendix was visualized to be dilated. Acellular myxoglobulosis material was protruding from both the lumen of the appendix and cecum (Figure 2). Cecal involvement was due to a mucin‐filled cecal wall sac contiguous with the appendiceal lumen; created as mucin seeped into the submucosa wall of the cecum. Histopathology diagnosed a low‐grade appendiceal mucinous neoplasm pT3Nx without cellular mural invasion or desmoplasia, and no extension beyond the mesoappendiceal margin (Figure 3). Screening tests for tumor markers CA 19‐9, CA‐125, and CEA were normal. Computed tomography (CT) and magnetic resonance imaging (MRI) scans of the abdomen and pelvis did not show signs of peritoneal involvement. Terminal ileal and colon biopsies were unremarkable, and the resected specimen of the cecum and appendix showed no signs of irritable bowel disease. Symptoms including weight loss, diarrhea, nausea, and pain all resolved after surgical resection. The patient will undergo abdominal and pelvic CT scans and tumor marker tests every 6 months for the next 2 years, then yearly thereafter to monitor for the potential development of PMP, a condition characterized by the spread of mucinous material throughout the abdominal cavity.^3^, ^5^, ^6^

## 2 DISCUSSION

The nomenclature of appendiceal mucinous neoplasms remains controversial and unclear. The Peritoneal Surface Oncology Group International in 2016 divided epithelial mucinous neoplasms of the appendix into the following histopathologic types: serrated polyp, low‐grade appendiceal mucinous neoplasm (LAMN), high‐grade appendiceal mucinous neoplasm (HAMN), and mucinous adenocarcinoma (with or without signet ring cells).^4^, ^5^ HAMNs and LAMNs have a few histologic features in common; however, HAMNs have more severe cytologic atypia. LAMNs and HAMNs characteristic histologic features include “pushing invasion.” This invasion can cause rupture of the appendiceal wall and lead to the spreading of mucin throughout the peritoneum.^5^

Laboratory findings for appendiceal mucinous neoplasms can include elevations in the tumor markers CEA, CA 19‐9, or CA‐125. In addition, a complete blood count may show signs of anemia.^7^ Abdominal CT, MRI, and ultrasound (US) are all potential scans that may detect an appendiceal mucocele, but they do not provide definitive information on the potential of malignancy.^6^ On colonoscopy, appendiceal mucoceles can normally be visualized as a smooth bulb protruding from the appendiceal orifice. Pathological findings include a normal appearing, grossly dilated appendix due to mucinous accumulation, hyalinization, and fibrosis of appendiceal walls.^8^

Treatment and prognosis for appendiceal neoplasms depend on the histopathologic characteristics of the tumor, tumor‐nodes‐metastasis stage, tumor grade, and presence of peritoneal disease. Treatment guidelines are not well defined and remain controversial regarding surgical approach and follow‐up surveillance. LAMNs that are localized to the appendix and consist of acellular mucin have an excellent prognosis after standard appendectomy due to a decreased likelihood of recurrence and less chance of developing into PMP.^1^, ^4^, ^6^ Formal right hemicolectomy has been proposed in patients with high‐risk features.^9^ In our case, positive appendectomy margins were achieved and the tumor consisted of acellular mucin, resulting in a more favorable outlook.

PMP is an indolent, but relentless accumulation of intra‐abdominal mucin resulting in gelatinous ascites.^5^ PMP development is usually the result of ruptured appendiceal mucinous tumors including LAMNs, HAMNs, and mucinous adenocarcinomas.^4^ The current standard treatment for PMP is cytoreduction and hyperthermic intraperitoneal chemotherapy. Prognosis depends on the specific subtype and histopathologic features.^3^, ^4^, ^5^ Many consider laparoscopy a safe and effective technique for removing appendiceal neoplasms. If possible, it is important to avoid mucin extrusion during removal.^7^

Appendiceal neoplasms have no clear direct association with inflammatory bowel disease^10^; however, they have been linked to other tumors of the ovary, endometrium, breast, kidney, colon, rectum, and liver in as many as 30% of cases.^6^ Surveillance includes abdominopelvic CT and tumor markers. Duration of surveillance can be 6 months for the first 2 years, followed by yearly for the next 5–10 years.^9^ Our patient's follow‐up care was determined by these surveillance guidelines.

This case highlights the diagnostic challenges and complex management of appendiceal mucinous neoplasms, especially in pediatric patients where they are extremely rare. Treatment decisions depend on histopathological factors, with surgery often playing a key role. Laparoscopy is a promising approach but requires careful handling to prevent mucin spread. The association with other malignancies also underscores the need for vigilant long‐term surveillance. Clear guidelines and a multidisciplinary approach are essential to navigate these complex cases and improve outcomes.

## CONFLICT OF INTEREST STATEMENT

The authors declare no conflict of interest.

## ETHICS STATEMENT

No research ethics board approval was required; however, informed consent from the parents was obtained to publish this work.
